# Optimized medium via statistical approach enhanced threonine production by *Pediococcus pentosaceus* TL-3 isolated from Malaysian food

**DOI:** 10.1186/s12934-019-1173-2

**Published:** 2019-07-22

**Authors:** Ye Heng Lim, Hooi Ling Foo, Teck Chwen Loh, Rosfarizan Mohamad, Raha Abdul Rahim, Zulkifli Idrus

**Affiliations:** 1 Institute of Bioscience, Universiti Putra Malaysia, 43400 UPM Serdang, Selangor Malaysia; 2 Department of Bioprocess Technology, Faculty of Biotechnology and Biomolecular Sciences, Universiti Putra Malaysia, 43400 UPM Serdang, Selangor Malaysia; 3 Department of Animal Science, Faculty of Agriculture, Universiti Putra Malaysia, 43400 UPM Serdang, Selangor Malaysia; 4 Institute of Tropical Agriculture and Food Security, Universiti Putra Malaysia, 43400 UPM Serdang, Selangor Malaysia; 5 Institute of Tropical Forestry and Forest Products, Universiti Putra Malaysia, 43400 UPM Serdang, Selangor Malaysia; 6 Department of Cell and Molecular Biology, Faculty of Biotechnology and Biomolecular Sciences, Universiti Putra Malaysia, 43400 UPM Serdang, Selangor Malaysia; 7 Halal Products Research Institute, Universiti Putra Malaysia, 43400 UPM Serdang, Selangor Malaysia

**Keywords:** Central composite design, *Pediococcus pentosaceus* TL-3, Plackett–Burman design, Statistical optimization, Threonine production

## Abstract

**Background:**

Threonine is an essential amino acid that is extensively used in livestock industry as feed supplement due to its pronounced effect in improving the growth performance of animals. Application of genetically engineered bacteria for amino acid production has its share of controversies after eosinophils myalgia syndrome outbreak in 1980s. This has urged for continuous search for a food grade producer as a safer alternative for industrial amino acid production. Lactic acid bacteria (LAB) appear as an exceptional candidate owing to their non-pathogenic nature and reputation of Generally Recognized as Safe (GRAS) status. Recently, we have identified a LAB, *Pediococcus pentosaceus* TL-3, isolated from Malaysian food as a potential threonine producer. Thus, the objective of this study was to enhance the threonine production by *P. pentosaceus* TL-3 via optimized medium developed by using Plackett–Burman design (PBD) and central composite design (CCD).

**Results:**

Molasses, meat extract, (NH_4_)_2_SO_4_, and MnSO_4_ were identified as the main medium components for threonine production by *P. pentosaceus* TL-3. The optimum concentration of molasses, meat extract, (NH_4_)_2_SO_4_ and MnSO_4_ were found to be 30.79 g/L, 25.30 g/L, 8.59 g/L, and 0.098 g/L respectively based on model obtained in CCD with a predicted net threonine production of 123.07 mg/L. The net threonine production by *P. pentosaceus* TL-3 in the optimized medium was enhanced approximately 2 folds compared to the control.

**Conclusions:**

This study has revealed the potential of *P. pentosaceus* TL-3 as a safer alternative to produce threonine. Additionally, the current study has identified the key medium components affecting the production of threonine by *P. pentosaceus* TL-3, followed by optimization of their concentrations by means of statistical approach. The findings of this study could act as a guideline for the future exploration of amino acid production by LAB.

## Background

Threonine is an essential amino acid that is used extensively in livestock industry as feed supplement due to its pronounced effect in improving the growth performance of animals [1]. It is the third limiting amino acid for poultry, after lysine and methionine. Threonine was deemed to play a vital role in maintenance of the proper gut function and essential for mucin synthesis [2]. Apart from that, supplementation of threonine was found effective in improving the breast meat deposition in broiler [3], as well as enhancing the intestinal morphology and growth performance of pigs [4] and starter Pekin ducks [5].

Currently, threonine production relies heavily on fermentation method by using modified strains of *Corynebacterium glutamicum* [6] and *Escherichia coli* [7]. However, pathogenicity of these microorganisms could raise concern for the consumers, whereby utilization of genetically engineered bacteria for amino acid production has its share of controversies particularly after the eosinophils myalgia syndrome (EMS) outbreak in 1980s attributed to the toxin produced by an engineered bacterium [8]. This has urged for continuous search of a safer alternative food grade amino acid producer. Lactic acid bacteria (LAB) appear as an exceptional candidate owing to their non-pathogenic nature and reputation as Generally Recognized as Safe (GRAS) microorganism [9]. Moreover, numerous studies have reported that LAB possess capability to produce various amino acids [10–18]. Nevertheless, there were limited documentation on the application of LAB for amino acid production.

Process optimization is an important step in industrial production processes to ensure higher productivity at minimal cost. Improvement of microbial metabolites production in a fermentation process is often achieved by manipulating the physical and nutritional parameters or via strain improvement. Optimization of nutritional parameter is one of the most prevalent approach due to its effectiveness [19]. Optimization of medium formulation can be accomplished by using either conventional or statistical method or combination of both. Conventional method involves varying an independent variable at a time while keeping the other variables constant. This is often laborious and time consuming especially when a huge number of variables are involved. In contrast, statistical method is rapid, more reliable and cost effective as it reduces the number of experimental runs tremendously [20, 21].

Dozens of designs are available in the statistical method and the choice is often dependent on the objectives of the experiment. Generally, they can be categorized into 3 main types, which are screening, factorial, and response surface methodology. Screening designs are also referred as Resolution III designs, which are commonly employed to scout the experimental space when the understanding on the system is limited. Plackett–Burman design (PBD) is an example of screening design, which is useful to elucidate the main effect of each variable [22]. It involves 4*n* experiments and the maximum number of variables that can be studied is 4*n* − 1, where a PBD with 12 experimental runs can handle up to 11 variables [23]. Meanwhile, central composite design (CCD) is one of the most commonly employed Response Surface Methodology. It comprises of three distinct parts including a factorial portion, a center point portion and an axial portion, which together serves to acquire comprehensive information and to determine the optimum operating settings.

Over the past decades, the studies on optimization of cultural conditions for threonine production were focused on *E. coli* and *C. glutamicum*. Despite optimization of glutamate [16] and GABA production [24, 25] by LAB had been conducted, there was no available report on optimization of medium formulation for threonine production by LAB. However, in our previous study, we have reported that *Pediococcus pentosaceus* TL-3 has the capability to produce various amino acids with exceptional ability to produce threonine and it was identified as a potential threonine producer [26]. Hence, the objectives of this study were to elucidate the effects of medium components on threonine production by *P. pentosaceus* TL-3 using PBD, followed by further optimization of the medium formulation by using CCD.

## Results and discussion

### Plackett–Burman design

The PBD is a useful tool for quick identification of key factors in a multivariable study [27]. In the current study, the effects of 22 medium components on threonine production by *P. pentosaceus* TL-3 were evaluated by using PBD with each medium component represented in two levels as shown in Table 1. A dummy variable (X) that has no chemical meaning was included in the PBD in order to determine the interactions between the variables [23]. The PBD matrix constituted of 24 experimental runs and their corresponding threonine production and cell population are presented in Table 2. The highest threonine production was detected in Run 5 (56.82 mg/L), followed by Run 4 (42.84 mg/L) and Run 13 (41.26 mg/L). However, the net threonine produced was achieved in the designed media and was significantly lower (p < 0.05) as compared to control (63.30 mg/L). This implied that further optimization was required in order to enhance the threonine production by *P. pentosaceus* TL-3.

**Table 1:** Coded and real values of medium components selected in PBD

Variables	Symbol code	Unit	Coded values	
			− 1	+ 1
Glucose	A	g/L	0	20
Sucrose	B	g/L	0	17.69
Fructose	C	g/L	0	19.08
Lactose	D	g/L	0	18.86
Molasses	E	g/L	0	25.08
Yeast extract	F	g/L	0	4
Peptone	G	g/L	0	10
Meat extract	H	g/L	0	8
K_2_HPO_4_	J	g/L	0	2
KH_2_PO_4_	K	g/L	0	2
Urea	L	g/L	0	3
NH_4_NO_3_	M	g/L	0	5
(NH_4_)_2_SO_4_	N	g/L	0	5
(NH_4_)_2_HC_6_H_5_O_7_	O	g/L	0	2
NaOAc	P	g/L	0	5
MgSO_4_	Q	g/L	0	0.2
MnSO_4_	R	g/L	0	0.04
Tween 80	S	mL/L	0	1
FeSO_4_	T	g/L	0	0.01
ZnSO_4_	U	g/L	0	0.01
CuSO_4_	V	g/L	0	0.01
Biotin	W	g/L	0	0.06

**Table 2:** PBD matrix for 22 medium components with coded values and their corresponding net threonine produced and cell population

Std run	A	B	C	D	E	F	G	H	J	K	L	M	N	O	P	Q	R	S	T	U	V	W	X	Net threonine produced (mg/L)	Cell population (log CFU/mL)
1	1	1	1	1	1	− 1	1	− 1	1	1	− 1	− 1	1	1	− 1	− 1	1	− 1	1	− 1	− 1	− 1	− 1	29.95 ± 0.99^F^	8.50 ± 0.02^N^
2	− 1	1	1	1	1	1	− 1	1	− 1	1	1	− 1	− 1	1	1	− 1	− 1	1	− 1	1	− 1	− 1	− 1	0.00 ± 0.00^N^	9.44 ± 0.03^B^
3	− 1	− 1	1	1	1	1	1	− 1	1	− 1	1	1	− 1	− 1	1	1	− 1	− 1	1	− 1	1	− 1	− 1	12.39 ± 0.29^K^	9.20 ± 0.02^EF^
4	− 1	− 1	− 1	1	1	1	1	1	− 1	1	− 1	1	1	− 1	− 1	1	1	− 1	− 1	1	− 1	1	− 1	42.84 ± 1.23^C^	8.89 ± 0.03^K^
5	− 1	− 1	− 1	− 1	1	1	1	1	1	− 1	1	− 1	1	1	− 1	− 1	1	1	− 1	− 1	1	− 1	1	56.82 ± 1.44^B^	9.24 ± 0.01^DE^
6	1	− 1	− 1	− 1	− 1	1	1	1	1	1	− 1	1	− 1	1	1	− 1	− 1	1	1	− 1	− 1	1	− 1	39.72 ± 0.96^D^	9.03 ± 0.02^H^
7	− 1	1	− 1	− 1	− 1	− 1	1	1	1	1	1	− 1	1	− 1	1	1	− 1	− 1	1	1	− 1	− 1	1	27.97 ± 0.79^G^	8.59 ± 0.01^M^
8	1	− 1	1	− 1	− 1	− 1	− 1	1	1	1	1	1	− 1	1	− 1	1	1	− 1	− 1	1	1	− 1	− 1	14.69 ± 0.46^J^	9.00 ± 0.01^HI^
9	− 1	1	− 1	1	− 1	− 1	− 1	− 1	1	1	1	1	1	− 1	1	− 1	1	1	− 1	− 1	1	1	− 1	0.00 ± 0.00^N^	7.45 ± 0.00^Q^
10	− 1	− 1	1	− 1	1	− 1	− 1	− 1	− 1	1	1	1	1	1	− 1	1	− 1	1	1	− 1	− 1	1	1	0.00 ± 0.00^N^	7.91 ± 0.01^O^
11	1	− 1	− 1	1	− 1	1	− 1	− 1	− 1	− 1	1	1	1	1	1	− 1	1	− 1	1	1	− 1	− 1	1	4.30 ± 0.05^M^	8.97 ± 0.03^IJ^
12	1	1	− 1	− 1	1	− 1	1	− 1	− 1	− 1	− 1	1	1	1	1	1	− 1	1	− 1	1	1	− 1	− 1	7.60 ± 0.15^L^	8.72 ± 0.01^L^
13	− 1	1	1	− 1	− 1	1	− 1	1	− 1	− 1	− 1	− 1	1	1	1	1	1	− 1	1	− 1	1	1	− 1	41.26 ± 0.89^CD^	9.24 ± 0.01^DE^
14	− 1	− 1	1	1	− 1	− 1	1	− 1	1	− 1	− 1	− 1	− 1	1	1	1	1	1	− 1	1	− 1	1	1	14.06 ± 0.28^JK^	8.60 ± 0.00^M^
15	1	− 1	− 1	1	1	− 1	− 1	1	− 1	1	− 1	− 1	− 1	− 1	1	1	1	1	1	− 1	1	− 1	1	16.84 ± 0.35^I^	9.28 ± 0.01^D^
16	1	1	− 1	− 1	1	1	− 1	− 1	1	− 1	1	− 1	− 1	− 1	− 1	1	1	1	1	1	− 1	1	− 1	8.71 ± 0.23^L^	9.10 ± 0.01^G^
17	− 1	1	1	− 1	− 1	1	1	− 1	− 1	1	− 1	1	− 1	− 1	− 1	− 1	1	1	1	1	1	− 1	1	30.76 ± 0.73^F^	8.97 ± 0.01^IJ^
18	1	− 1	1	1	− 1	− 1	1	1	− 1	− 1	1	− 1	1	− 1	− 1	− 1	− 1	1	1	1	1	1	− 1	17.66 ± 0.25^HI^	8.51 ± 0.01^N^
19	− 1	1	− 1	1	1	− 1	− 1	1	1	− 1	− 1	1	− 1	1	− 1	− 1	− 1	− 1	1	1	1	1	1	17.98 ± 0.50^HI^	8.90 ± 0.02^K^
20	1	− 1	1	− 1	1	1	− 1	− 1	1	1	− 1	− 1	1	− 1	1	− 1	− 1	− 1	− 1	1	1	1	1	14.04 ± 0.22^JK^	8.93 ± 0.02^JK^
21	1	1	− 1	1	− 1	1	1	− 1	− 1	1	1	− 1	− 1	1	− 1	1	− 1	− 1	− 1	− 1	1	1	1	8.36 ± 0.28^L^	9.16 ± 0.01^F^
22	1	1	1	− 1	1	− 1	1	1	− 1	− 1	1	1	− 1	− 1	1	− 1	1	− 1	− 1	− 1	− 1	1	1	35.50 ± 0.71^E^	9.38 ± 0.01^C^
23	1	1	1	1	− 1	1	− 1	1	1	− 1	− 1	1	1	− 1	− 1	1	− 1	1	− 1	− 1	− 1	− 1	1	19.37 ± 0.55^H^	8.99 ± 0.01^HI^
24	− 1	− 1	− 1	− 1	− 1	− 1	− 1	− 1	− 1	− 1	− 1	− 1	− 1	− 1	− 1	− 1	− 1	− 1	− 1	− 1	− 1	− 1	− 1	0.00 ± 0.00^N^	7.73 ± 0.02^P^
MRS	1	− 1	− 1	− 1	− 1	1	1	1	1	− 1	− 1	− 1	− 1	1	1	1	1	1	− 1	− 1	− 1	− 1	− 1	63.30 ± 2.10^A^	9.57 ± 0.01^A^

Values are mean ± SEM, n = 3. Mean ± SEM within the same column that share a common superscript are not significantly different (p > 0.05)

The adequacy of the model and the significance of the medium components on threonine production by *P. pentosaceus* TL-3 were evaluated by ANOVA (Table 3). The statistical significance of the model was evaluated by Fisher’s statistical test (F-test). The *p* value of the model (0.0474) inferred that the model was significant and there was merely 4% of chance that the F-value of the model this large could occur due to noise. In addition, the coefficient of determination, R^2^ of the model was 0.9998, implying that the model could explain 99% of the variation in response. Moreover, the “predicted R^2^” (0.9055) was in reasonable agreement with the “adjusted R^2^” (0.9962), where the difference was less than 0.2, indicating that the model resembles good fitness. The closer the R^2^ value to 1.0, the better the correlation between the experimental and predicted values [28]. Furthermore, the adequate precision value of the present model (62.573) was greater than 4, suggesting that the model could be used to navigate the design space.

**Table 3:** ANOVA of PBD for the effects of medium components on threonine production by *P. pentosaceus* TL-3

Source	Sum of squares	df	Mean square	F value	p-value Prob > F	
Model	5798.03	22	263.55	277.11	0.05	Significant
A—Glucose	24.01	1	24.01	25.25	0.13	
B—Sucrose	3.52	1	3.52	3.71	0.30	
D—Lactose	389.05	1	389.05	409.07	0.03	Significant
E—Molasses	18.70	1	18.70	19.66	0.14	
F—Yeast extract	360.50	1	360.50	379.06	0.03	Significant
G—Peptone	1500.00	1	1500.00	1577.21	0.02	Significant
H—Meat extract	1619.60	1	1619.60	1702.97	0.02	Significant
J—K_2_HPO_4_	121.08	1	121.08	127.31	0.06	
K—KH_2_PO_4_	7.93	1	7.93	8.34	0.21	
L—Urea	347.43	1	347.43	365.31	0.03	Significant
M—NH_4_NO_3_	2.17	1	2.17	2.28	0.37	
N—(NH_4_)_2_SO_4_	182.20	1	182.20	191.58	0.05	Significant
O—(NH_4_)_2_HC_6_H_5_O_7_	1.20	1	1.20	1.26	0.46	
P—NaOAc	56.44	1	56.44	59.34	0.08	
Q—MgSO_4_	35.85	1	35.85	37.70	0.10	
R—MnSO_4_	747.64	1	747.64	786.12	0.02	Significant
S—Tween 80	70.28	1	70.28	73.90	0.07	
T—FeSO_4_	58.87	1	58.87	61.90	0.08	
U—ZnSO_4_	164.91	1	164.91	173.40	0.05	Significant
V—CuSO_4_	15.50	1	15.50	16.30	0.15	
W—Biotin	21.58	1	21.58	22.69	0.13	
X—dummy	49.58	1	49.58	52.14	0.09	
Residual	0.95	1	0.95			
Cor total	5798.98	23				

R^2^: 0.9998; Adj R^2^: 0.9962; Pred R^2^: 0.9055; Adeq precision: 62.573

Based on the ANOVA of threonine production (Table 3), 8 of the medium components including lactose (D), yeast extract (F), peptone (G), meat extract (H), urea (L), (NH_4_)_2_SO_4_ (N), MnSO_4_ (R), and ZnSO_4_ (U) were affected significantly (p < 0.05) the production of threonine by *P. pentosaceus* TL-3, whereas the other medium components did not contribute significantly (p > 0.05) to threonine production. Furthermore, the insignificance of dummy variable (p > 0.05) suggested the possible absence of significant interactions between the variables [23]. Equation 1 expresses the production of threonine (Y) by *P. pentosaceus* TL-3 in terms of medium components, where the coded symbols (A–X) are as described in Table 1.

1 $$\begin{aligned} {\text{Y}} & = 19.06 - 1.00{\text{A }} - 0.38{\text{B }} - 4.03{\text{D }} + 0.88{\text{E }} + 3.88{\text{F }} + 7.91{\text{G }} \hfill \\ & \quad + 8.21{\text{H}} + 2.25{\text{J }} - 0.57{\text{K }} - 3.80{\text{L }} - 0.30{\text{M }} + 2.76{\text{N }} + 0.22{\text{O }} \hfill \\ & \quad - 1.53{\text{P }} - 1.22{\text{Q}} + 5.58{\text{R }} - 1.71{\text{S }} + 1.57{\text{T }} - 2.62{\text{U }} + 0.80{\text{V }} \hfill \\ & \quad + 0.95{\text{W }} + 1.44{\text{X}} \hfill \\ \end{aligned}$$

**Fig. 1:**
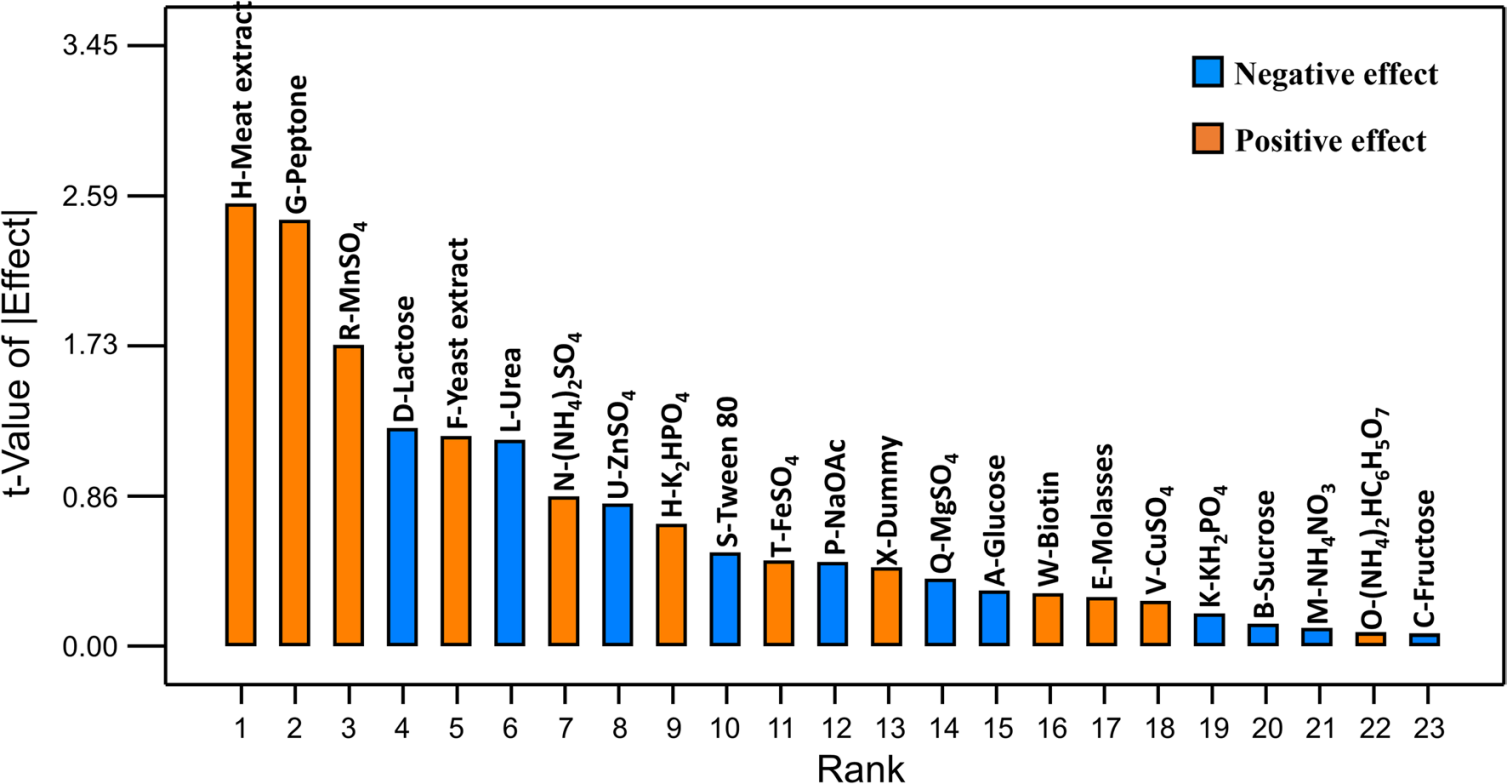
Pareto chart of the effects of medium components on threonine production by *P. pentosaceus* TL-3

Meanwhile, Fig. 1 depicts the effects of medium components on the production of threonine by *P. pentosaceus* TL-3. Out of the 22 studied medium components, half of the medium components including meat extract, peptone, MnSO_4_, yeast extract, (NH_4_)_2_SO_4_, K_2_HPO_4_, FeSO_4_, biotin, molasses, CuSO_4_, and (NH_4_)_2_HC_6_H_5_O_7_ demonstrated positive effect on threonine production, while the other half medium components exerted negative effect on threonine production. A positive effect indicates that increased level of the medium component promotes the threonine production and vice versa [29]. Among the 11 medium components that demonstrated positive effect on threonine production, 5 of them including yeast extract, peptone, meat extract, (NH_4_)_2_SO_4_, and MnSO_4_ were significant at p-value less than 0.05 (Table 3). In contrast, out of the 11 medium components with negative effect, 3 of them including lactose, urea, and ZnSO_4_ were significant at p-value less than 0.05.

Among the tested carbon sources (glucose, sucrose, fructose, lactose and molasses), only molasses exerted a positive effect on threonine production despite the effect was not significant (p > 0.05), whereas the other tested carbon sources demonstrated a negative effect on threonine production with lactose showing the highest inhibitory effect. Molasses had been widely utilized as an alternative carbon source for fermentative production of various amino acids [30] and its stimulatory effect was well reported. Moreover, Hagino and Nakayama [31] reported that molasses was the best carbon source for tryptophan production by *Corynebacterium*. Apart from acting as a carbon source, molasses contains abundant amount of vitamin such as niacin (vitamin B_3_) [32], which is the precursor of nicotinamide adenine dinucleotide phosphate (NADP^+^) [33] that acts as coenzyme for numerous enzymes involved in the biosynthesis of threonine. For instances, the enzymes β-aspartate semialdehyde dehydrogenase and homoserine dehydrogenase require NADP^+^ as a coenzyme for the conversion of aspartyl-β-phosphate into β-aspartate-semialdehyde [34] and the synthesis of homoserine from β-aspartate-semialdehyde [35, 36] respectively. Additionally, molasses also contains ample amount of pyridoxal phosphate (vitamin B_6_) [37, 38], which acts as a coenzyme for threonine synthase involving in the synthesis of threonine from phosphohomoserine [39].

Meanwhile, all the studied organic nitrogen sources (yeast extract, peptone, meat extract) exerted a positive effect on threonine production with meat extract exhibiting the most prominent effect, followed by peptone and yeast extract. Production of various amino acids by LAB using media containing organic nitrogen sources such as yeast extract, peptone or meat extract was well documented [15, 24, 40]. In addition, yeast extract was also employed for amino acid production by *E. coli* [41, 42]. The significant effect of organic nitrogen sources, particularly meat extract on threonine production by *P. pentosaceus* TL-3 may due to the presence of abundant coenzymes in meat extract that are essential for enzymes involved in biosynthesis of threonine. Meat extract is rich in vitamin B_3_ and vitamin B_6,_ which are essential for NADPH and pyridoxal phosphate biosynthesis that act as a coenzyme for β-aspartate semialdehyde dehydrogenase, homoserine dehydrogenase and threonine synthase enzymes respectively. Moreover, the organic nitrogen sources could be a source of aspartate, which is the precursor for threonine synthesis [43].

Amongst the 4 studied inorganic nitrogen sources, urea and (NH_4_)_2_SO_4_ affected threonine production significantly (p < 0.05), whereby urea exerted a negative effect and (NH_4_)_2_SO_4_ demonstrated a positive effect. Davati et al. [44] has reported the use of urea for glutamate production by *Corynebacterium*, whereas (NH_4_)_2_SO_4_ has been used for threonine production by *Serratia marcescens* [45] and *E. coli* [42, 46–48]. Furthermore, Miyajima and Shiio [49] reported that inorganic nitrogen source could affect the activity of homoserine kinase enzyme, which is responsible for the conversion of homoserine into phosphohomoserine. The homoserine kinase activity was increased by 2-folds when (NH_4_)_2_SO_4_ was added in the growth medium.

In comparison, 2 out of the 8 tested mineral sources demonstrated a significant effect (p < 0.05) on threonine production by *P. pentosaceus* TL-3, whereby MnSO_4_ displayed a significant positive effect (p < 0.05) and ZnSO_4_ exhibited a significant negative effect (p < 0.05). However, the other metal ions (K^+^, Na^+^, Mg^2+^, Fe^2+^, Cu^2+^) did not contribute significantly (p > 0.05) to threonine production. Metal ions may affect the bacterial metabolic activity due to specific ionic and water binding capacity [50]. The pronounced effect of Mn^2+^ on the production of LAB metabolites was well documented [51, 52]. Furthermore, the use of medium containing Mn^2+^ for gamma-aminobutyric acid (GABA) production by LAB [24] and threonine production by *E. coli* [42] were also reported. The significant effect of Mn^2+^ on threonine production might be due to its role as a cofactor for the enzymes involved in the biosynthesis of threonine. For instances, divalent cation such as Mg^2+^, Mn^2+^ or Co^2+^ act as the physiological activator of the first enzyme involves in biosynthesis of threonine, aspartokinase, which is responsible for the phosphorylation of aspartate into aspartyl-ß-phosphate [53]. Moreover, the role of divalent cations as a cofactor for the enzyme homoserine kinase was also well documented. Miyajima and Shiio [49] reported that Mg^2+^ was essential for the enzyme homoserine kinase, whereby the enzymatic activity was not detected when Mg^2+^ was absent. In addition, they also discovered that Mn^2+^ could potentially replace Mg^2+^ as a cofactor for homoserine kinase, whereby similar level of enzyme activity was detected in the presence of either Mg^2+^ or Mn^2+^ ions. The stimulatory effect of divalent cations on the enzymatic activity of homoserine kinase was also well documented by other researchers [54, 55].

Contradictory findings were obtained for both vitamin (biotin) and non-ionic surfactant (Tween 80) in current study, whereby both elements did not exert significant effect (p > 0.05) on the production of threonine by *P. pentosaceus* TL-3. Other researchers reported that biotin was often crucial for amino acid production by *E. coli* [47] and *C. glutamicum* [56]. In the present study, biotin did not contribute significantly (p > 0.05) to the production of threonine by *P. pentosaceus* TL-3, which was probably due to the use of different producer strains as compared to other studies. Another possible explanation might be the requirement of these coenzymes are often in traces amount. Hence, the use of molasses that contains ample amount of biotin [57] would be sufficient to stimulate the production of threonine by *P. pentosaceus* TL-3. On the other hand, previous studies revealed that Tween 80 was one of the key factors affecting the production of LAB metabolites. Inclusion of Tween 80 in the growth medium enhanced the bacteriocin production by more than 50% [58], whereby Tween 80 that acted as non-ionic surfactant has facilitated the secretion of metabolites by altering the membrane fluidity of bacteria cells [59]. Despite numerous reports suggested the important role of Tween 80 on the production of LAB metabolites, yet there was limited documentation regarding the role of Tween 80 on amino acid production. However, Tween 80 has been identified as one of the key factors affecting GABA production by *Lactobacillus brevis* NCL912 [24] and glutamate production by *Brevibacterium* sp. [60].

The effects of medium components on the growth of *P. pentosaceus* TL-3 were also analyzed in the 24 experimental runs of PBD. The design matrix of the PBD and their respective cell population are presented in Table 2. Results obtained in the current study showed that *P. pentosaceus* TL-3 was able to grow well in most of the designed media, whereby the cell population was exceeding 8.5 log CFU/mL in most of the experimental runs, except for run 9, 10 and 24; which exhibited a decreased cell population. One of the similarities among run 9, 10 and 24 was the absence of organic nitrogen sources (peptone, meat extract, yeast extract), implying that organic nitrogen is crucial for the survival of LAB. This is in line with the findings reported by Saeed and Salam [61], where LAB is a fastidious microorganism and unable to grow on simple mineral media supplemented with carbon source solely. They require organic nitrogen sources in order to grow well. The highest cell population was recorded in run 2 which constituted of sucrose, fructose, lactose, molasses, yeast extract, meat extract, KH_2_PO_4_, urea, (NH_4_)_2_HC_8_H_6_O_7_, NaOAc, Tween 80 and ZnSO_4_ with 9.44 log CFU/mL, followed by run 22 with 9.38 log CFU/mL. Yet the cell population detected in run 2 was still significantly lower (p < 0.05) as compared to the control that recorded 9.57 log CFU/mL of cell population.

The adequacy of the model and significance of the medium components on the growth of *P. pentosaceus* TL-3 were evaluated by ANOVA (Table 4). The statistical significance of the model was evaluated by F-test. The p-value of the model (0.01) inferred that the model was significant, and the occurrence of the F-value attributed to the noise was as low as 1%. In addition, the R^2^ value of the model was highly close to 1, indicating the strong predictive strength of the model. Furthermore, the “adjusted R^2^” value (0.9997) and “predicted R^2^” value (0.9918) of the model were in reasonable agreement, whereby the difference is less than 0.2, indicating that the model displayed good fitness. Moreover, the adequate precision value of the model was 221.14, suggesting a strong signal compared to noise ratio and the model could be used to navigate the design space.

**Table 4:** ANOVA of PBD for the effects of medium components on the growth of *P. pentosaceus* TL-3

Source	Sum of squares	df	Mean square	F value	p-value Prob > F	
Model	5.99	22	0.27	3197.50	0.01	Significant
A—Glucose	0.48	1	0.48	5684.23	< 0.01	Significant
B—Sucrose	0.05	1	0.05	637.72	0.03	Significant
C—Fructose	0.11	1	0.11	1314.61	0.02	Significant
E—Molasses	0.44	1	0.44	5176.97	< 0.01	Significant
F—Yeast extract	1.81	1	1.81	21277.84	< 0.01	Significant
G—Peptone	0.14	1	0.14	1670.85	0.02	Significant
H—Meat extract	1.15	1	1.15	13517.33	< 0.01	Significant
J—K_2_HPO_4_	0.02	1	0.02	215.44	0.04	Significant
K—KH_2_PO_4_	0.09	1	0.09	1018.95	0.02	Significant
L—Urea	< 0.01	1	< 0.01	13.56	0.17	
M—NH_4_NO_3_	0.04	1	0.04	419.49	0.03	Significant
N—(NH_4_)_2_SO_4_	0.62	1	0.62	7297.38	< 0.01	Significant
O—(NH_4_)_2_HC_6_H_5_O_7_	0.12	1	0.12	1400.94	0.02	Significant
P—NaOAc	0.16	1	0.16	1822.65	0.01	Significant
Q—MgSO_4_	0.11	1	0.11	1284.49	0.02	Significant
R—MnSO_4_	0.09	1	0.09	1093.48	0.02	Significant
S—Tween 80	0.06	1	0.06	750.79	0.02	Significant
T—FeSO_4_	0.02	1	0.02	198.42	0.05	Significant
U—ZnSO_4_	0.10	1	0.10	1144.21	0.02	Significant
V—CuSO_4_	0.09	1	0.09	1080.65	0.02	Significant
W—Biotin	0.10	1	0.10	1158.44	0.02	Significant
X—dummy	0.18	1	0.18	2166.55	0.01	Significant
Residual	< 0.01	1	< 0.01			
Cor Total	5.99	23				

R^2^: 1.0000; Adj R^2^: 0.9997; Pred R^2^: 0.9918; Adeq precision: 221.140

Based on the ANOVA table (Table 4), 20 out of the 22 studied medium components affected the growth of the isolate significantly (p < 0.05) and 5 of them (glucose, molasses, yeast extract, meat extract and (NH_4_)_2_SO_4_ were highly significant (p < 0.01). In contrast, urea and lactose did not contribute significantly to the growth of *P. pentosaceus* TL-3 (p > 0.05). Moreover, p-value of the dummy variable (0.01) implied that the interaction possibilities between the medium components, which warrant further investigation in subsequent experiment by using a higher resolution design [62]. The growth of *P. pentosaceus* TL-3 (Z) can be expressed by Eq. (2) and the coded symbols (A–X) are described in Table 1.

2 $$\begin{aligned} {\text{Z }} & = 8.82 + 0.14{\text{A }} + 0.05{\text{B }} + 0.07{\text{C }} + 0.14{\text{E }} + 0.27{\text{F }} + 0.08{\text{G}} \hfill \\ & \quad + 0.22{\text{H}} - 0.03{\text{J }} - 0.06{\text{K }} + 0.01{\text{L }} - 0.04{\text{M }} - 0.16{\text{N }} + 0.07{\text{O }} \hfill \\ & \quad + 0.08{\text{P }} + 0.07{\text{Q}} + 0.06{\text{R }} - 0.05{\text{S }} + 0.03{\text{T }} + 0.06{\text{U }} + 0.06{\text{V }} \hfill \\ & \quad - 0.06{\text{W }} + 0.09{\text{X}} \hfill \\ \end{aligned}$$

**Fig. 2:**
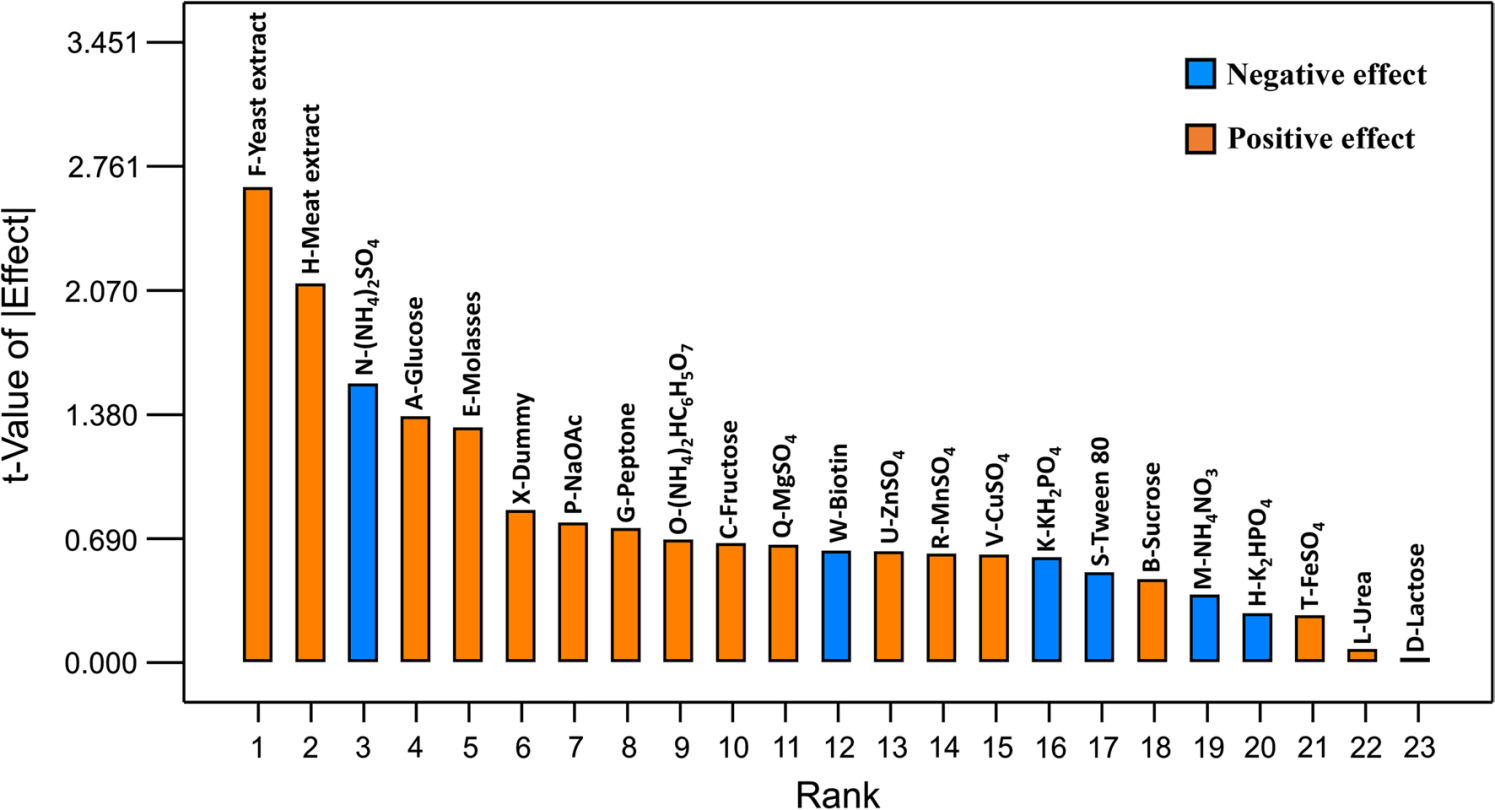
Pareto chart of the effect of variables on growth of *P. pentosaceus* TL-3

Figure 2 depicts the effects of medium components on the growth of *P. pentosaceus* TL-3, whereby majority of the studied medium components exerted a positive effect on the growth, except for (NH_4_)_2_SO_4_, biotin, KH_2_PO_4_, Tween 80, NH_4_NO_3_ and K_2_HPO_4_, which demonstrated a significant inhibitory effect (p < 0.05). Amongst the 16 medium components with positive effect, most of them were significant at p-value less than 0.05 (Table 4), except urea and lactose (p > 0.05). Furthermore, the tested carbon sources (glucose, sucrose, fructose, lactose and molasses) exerted a significant positive effect (p < 0.05) on the cell growth, except lactose, suggesting that the isolate could utilize a wide variety of carbon sources for its growth. This is in line with the findings reported previously, whereby LAB could utilize various carbon sources for their growth, including simple sugars such as glucose, sucrose, mannose and etcetera [63], as well as complex carbohydrates such as agricultural wastes [64] and their hydrolysate [65].

Similar trend was observed in the organic nitrogen sources, whereby the studied organic nitrogen sources (yeast extract, peptone, meat extract) exerted a significant positive effect (p < 0.05) on the growth of *P. pentosaceus* TL-3. The important role of organic nitrogen source on the growth of LAB has been well documented. LAB are nutritionally fastidious microorganisms and they often require exogenous supply of peptides or amino acid for growth and survival [66]. Rodrigues et al. [67] also reported that organic nitrogen sources were important elements for the growth of a probiotic bacterium, *Lactococcus lactis* 53. However, the organic nitrogen sources did not contribute significantly to the growth of *Streptococcus thermophilus* A [67], suggesting that the requirement of organic nitrogen sources for cell growth was species dependent, whereby different species of LAB has different preference on organic nitrogen source. Amongst the 3 organic nitrogen sources used in current study, yeast extract exerted the highest stimulatory effect on the growth of *P. pentosaceus* TL-3. The crucial role of yeast extract on the bacterial growth could be attributed to its rich vitamin B content, which is a vital element for the growth of LAB as reported by Kadam et al. [68].

In comparison, among the inorganic nitrogen sources tested in the current study, (NH_4_)_2_SO_4_ and NH_4_NO_3_ demonstrated a negative effect on the growth of *P. pentosaceus* TL-3, with (NH_4_)_2_SO_4_ being highly significant (p < 0.01), while NH_4_NO_3_ was significant at p-value less than 0.05. The negative effect of inorganic nitrogen revealed the inability of LAB to assimilate inorganic nitrogen [69]. In contrast, ammonium citrate [(NH_4_)_2_HC_6_H_5_O_7_] displayed a significant positive effect (p < 0.05) on the growth of *P. pentosaceus* TL-3. Contradictory results were reported by Rodrigues et al. [67], whereby (NH_4_)_2_HC_6_H_5_O_7_ did not influence the cell growth significantly, despite it contributed significantly on lactic acid production by *Lactobacillus amylophilus* GV6 [70]. In addition, urea also did not contribute significantly (p > 0.05) to the biomass formation of *P. pentosaceus* TL-3. This agrees with the findings reported by de Carvalho et al. [71], whereby addition of urea did not promote the growth of LAB.

Table 4 clearly shows that all the studied minerals including K_2_HPO_4_, KH_2_PO_4_, NaOAc, MgSO_4_, MnSO_4_, FeSO_4_, ZnSO_4_ and CuSO_4_ contributed significantly (p < 0.05) to the cell growth of *P. pentosaceus* TL-3. Majority of the studied mineral sources exhibited a stimulatory effect on the growth of the studied strain, except for K_2_HPO_4_ and KH_2_PO_4_, which affected the cell growth in a negative manner. The negative effect of K_2_HPO_4_ and KH_2_PO_4_ might be attributed to phosphate ions, which elevated the pH of the medium [72] and consequently retarded the growth of the acidophilic *P. pentosaceus* TL-3 that preferred acidic growing environment [73, 74]. In contrast, the positive effects of NaOAc might be due to the buffering capacity of acetate ions, which maintained the pH of the medium in slight acidic condition, thereby enhancing the growth of the acidophilic LAB [75]. In addition, Shi et al. [25] reported that the highest production of GABA by *Lactobacillus brevis* TCCC 13007 was noted under acidic condition (pH 4.5). Meanwhile, the stimulatory effect of other mineral ions (Mn^2+^, Mg^2+^, Fe^2+^, Cu^2+^) on LAB growth were well documented. The supplementation of mineral ions such as Mn^2+^, Mg^2+^, Ca^2+^ Fe^2+^, Co^2+^ and Cu^2+^ in the growth medium of LAB resulted in at least 2-fold increment of growth of all the tested LAB. However, addition of Zn^2+^ showed stimulatory effect on the growth of some of the studied LAB strains [76]. Additionally, stimulatory effect of Mn^2+^ on the growth of *Lactobacillus salivarius* CRL 1328 was also reported by Tomas et al. [75]. This was in line with the findings obtained in the present study, whereby all mineral ions (Mn^2+^, Mg^2+^, Fe^2+^, Cu^2+^, Zn^2+^) promoted the cell growth of *P. pentosaceus* TL-3 significantly (p < 0.05).

Interestingly, results obtained in the present study showed that the non-ionic surfactant (Tween 80) exhibited a significant (p < 0.05) inhibitory effect on the cell growth of *P. pentosaceus* TL-3. Oh et al. [77] has also discovered that Tween 80 did not contribute significantly to the growth of *Lactobacillus casei* YIT 9018. However, contradictory findings were reported by other researchers. The addition of Tween 80 enhanced the growth of LAB and other microorganisms [78, 79]. The stimulatory effect of Tween 80 could be due to the presence of oleic acid, which is a crucial growth factor for LAB [78]. Supplementation of Tween 80 allows the bacteria to incorporate oleic acid into the cell membrane, which is subsequently converted to cyclopropane fatty acids to increase the fluidity of LAB membranes, as well as to protect the LAB from various environmental stresses such as deleterious effect of oxygen, extreme pH and temperature [61]. As for the effect of vitamin, biotin demonstrated a significantly negative effect (p < 0.05) on the growth of *P. pentosaceus* TL-3. Contradictory finding was reported by Tripuraneni [80], who has reported that the supplementation of biotin enhanced the growth of various LAB strains. One of the possible explanations was biotin is often required in trace amount and the presence of abundant biotin in molasses is sufficient to sustain the growth of the bacteria [81]. Further increasing of the biotin concentration may hence promoted an adverse effect.

Based on the results obtained from Plackett–Burman experiment, a validation test constituted of 7 formulated media (Table 5) was conducted to verify the effects of medium components on threonine production by *P. pentosaceus* TL-3. The growth and net threonine production of *P. pentosaceus* TL-3 in different formulated media and MRS medium are presented in Table 6. MRS medium was used as a control. Generally, the net threonine production by using the 7 formulated media was ranging between 2.33 and 48.89 mg/L, which was significantly lower (p < 0.05) than the MRS control medium (60.10 mg/L). Amongst the 7 formulated media, the highest net threonine production was detected in Medium 1, which constituted of all the significant medium components. However, the net threonine production that achieved in Medium 1 was not significantly different (p > 0.05) as compared to Medium 3 and Medium 6 with approximately 47 mg/L of net threonine production. Furthermore, the highest cell growth was also detected in MRS control medium with 9.46 log CFU/mL, followed by Medium 4 with 9.33 log CFU/mL. However, Medium 6 was selected for further optimization study since the net threonine production was not significantly different (p > 0.05) as compared to Medium 1 and Medium 3. Moreover, Medium 6 contained the least medium components and hence the cost of the medium was the lowest among the 3 formulated media (Medium 1, 3 and 6) that resulted the highest net threonine production.

**Table 5:** Media formulation for validation of the effects of significant medium components on threonine production

Media formulation	Concentration (g/L)
Medium 1	
Meat extract	8
Peptone	10
MnSO_4_	0.04
Lactose	18.86
Yeast extract	4
Urea	3
(NH_4_)_2_SO_4_	5
ZnSO_4_	0.01
Medium 2	
Meat extract	8
Peptone	10
MnSO_4_	0.04
Yeast extract	4
(NH_4_)_2_SO_4_	5
Medium 3	
Meat extract	8
Peptone	10
MnSO_4_	0.04
Yeast extract	4
(NH_4_)_2_SO_4_	5
Molasses	25.08
Medium 4	
Meat extract	8
Peptone	10
MnSO_4_	0.04
Yeast extract	4
(NH_4_)_2_SO_4_	5
K_2_HPO_4_	2
FeSO_4_	0.01
Biotin	0.06
Molasses	25.08
CuSO_4_	0.01
(NH_4_)_2_HC_6_H_5_O_7_	2
Medium 5	
Molasses	25.08
Peptone	10
(NH_4_)_2_SO_4_	5
MnSO_4_	0.04
Medium 6	
Molasses	25.08
Meat extract	8
(NH_4_)_2_SO_4_	5
MnSO_4_	0.04
Medium 7	
Molasses	25.08
Yeast extract	4
(NH_4_)_2_SO_4_	5
MnSO_4_	0.04

**Table 6:** Growth and net threonine produced by *P. pentosaceus* TL-3 in different media

Media	Cell population (Log CFU/mL)	Net threonine produced (mg/L)
1	8.94 ± 0.02^D^	48.89 ± 5.57^B^
2	8.69 ± 0.05^F^	2.33 ± 1.24^D^
3	9.15 ± 0.01^C^	47.12 ± 3.33^B^
4	9.33 ± 0.01^B^	31.99 ± 4.06^C^
5	8.69 ± 0.02^F^	29.36 ± 1.97^C^
6	8.99 ± 0.02^D^	47.14 ± 0.62^B^
7	8.83 ± 0.05^E^	29.95 ± 0.20^C^
MRS	9.46 ± 0.01^A^	60.10 ± 4.04^A^

Values are mean ± standard error of the mean (SEM), n = 3. Mean ± SEM within the same column that share a different superscript are significantly different (p > 0.05)

### Steepest ascent

Molasses, meat extract, (NH_4_)_2_SO_4_ and MnSO_4_ were identified to be the most important medium components for threonine production by *P. pentosaceus* TL-3 and therefore they were selected for further optimization in this study. A steepest ascent experiment with 10 steps increment was used to determine the neighborhood of optimum concentration of these medium components. The high level (+ 1) of the medium components in the PBD was used as the origin of the steepest ascent experiment and the first-order model (Eq. 1) generated by the PBD was employed to determine the direction and step length of each medium component. Since the selected medium components exhibited a positive effect on threonine production by *P. pentosaceus* TL-3, increasing the concentration would increase the net threonine production. Hence, the concentration of medium components was moved along the path of steepest ascent. The coefficient of meat extract (the largest among the 4 medium components) was used as the standard for calculation of step length. Thus, the concentrations of molasses, (NH_4_)_2_SO_4_ and MnSO_4_ were increased by 5.5%, 17% and 34% respectively when the concentration of meat extract was increased by 50% as presented in Table 7.

**Table 7:** Steepest ascent for threonine production by *P. pentosaceus* TL-3

No.	Run	Medium compenet level, g/L			
		Molasses (A)	Meat extract (B)	(NH_4_)_2_SO_4_ (C)	MnSO_4_ (D)
	Δ	1.38	4	0.85	0.014
1	Origin	25.08	8	5.00	0.040
2	Origin + Δ	26.46	12	5.85	0.054
3	Origin + 2Δ	27.84	16	6.70	0.068
4	Origin + 3Δ	29.22	20	7.55	0.082
5	Origin + 4Δ	30.60	24	8.40	0.096
6	Origin + 5Δ	31.98	28	9.25	0.110
7	Origin + 6Δ	33.36	32	10.10	0.124
8	Origin + 7Δ	34.74	36	10.95	0.138
9	Origin + 8Δ	36.12	40	11.80	0.152
10	Origin + 9Δ	37.50	44	12.65	0.166
11	Origin + 10Δ	38.88	48	13.50	0.180

Table 8 presents the cell population and net threonine production by *P. pentosaceus* TL-3 in the steepest ascent experiment. Generally, the cell population increased progressively from run 1 (8.71 log CFU/mL) to run 6 (9.23 log CFU/mL). However, the reduction of cell population was noted when the concentration of medium components was increased further, indicating that higher concentration exerted inhibitory effect on the cell growth. Similar trend was observed in the threonine production, whereby the net threonine production was increased steadily from run 1 (45.42 mg/L) to run 5 (116.83 mg/L). The highest net threonine production was detected in run 5 with 30.6 g/L molasses, 24 g/L meat extract, 8.4 g/L (NH_4_)_2_SO_4_ and 0.096 g/L MnSO_4_. After 4 steps of increment, the net threonine production was improved by approximately 3 folds as compared to the origin (run 1). Moreover, the net threonine production achieved at run 5 was significantly higher (p < 0.05) as compared to the control (MRS medium). Inhibitory effect on threonine production by *P. pentosaceus* TL-3 was noted when the concentration of the medium components was increased further. Hence, the concentration of the medium components at run 5 was selected as the center point for further optimization in the subsequent CCD experiment.

**Table 8:** Cell population and net threonine produced by *P. pentosaceus* TL-3 in different media in steepest ascent experiment

Run	Cell population (log CFU/mL)	Net threonine produced (mg/L)
1	8.71 ± 0.02^F^	45.42 ± 0.11^H^
2	8.95 ± 0.01^E^	48.62 ± 0.10^G^
3	9.00 ± 0.01^D^	60.34 ± 0.91^F^
4	9.01 ± 0.02^D^	88.96 ± 0.36^C^
5	9.20 ± 0.01^B^	116.83 ± 0.29^A^
6	9.23 ± 0.01^B^	95.37 ± 0.14^B^
7	9.03 ± 0.01^D^	73.14 ± 2.14^D^
8	9.15 ± 0.01^C^	57.77 ± 0.71^F^
9	9.19 ± 0.01^B^	48.65 ± 0.40^G^
10	9.14 ± 0.01^C^	36.99 ± 0.84^I^
11	9.12 ± 0.01^C^	19.47 ± 0.16^J^
MRS	9.32 ± 0.01^A^	64.09 ± 2.46^E^

Values are mean ± standard error of the mean (SEM), n = 3. Mean ± SEM within the same column that share similar superscript are not significantly different (p > 0.05)

### Central composite design

Five levels of concentrations were assigned to each medium component as shown in Table 9. A 30 experimental runs CCD was suggested and their corresponding experimental and predicted net threonine production are presented in Table 10. The highest net threonine production was detected in run 25 to run 30, which represented the center point of the CCD with approximately 120 mg/L of net threonine produced, followed by run 16, which constituted of high level (+ 1) of all the 4 studied medium components with up to 100 mg/L of net threonine produced. The net threonine produced detected in run 25 to run 30 was significantly higher (p < 0.05) than the control (MRS medium), which recorded 60.47 mg/L of net threonine produced.

**Table 9:** Coded and real values of medium compenents for CCD of threonine production by *P. pentosaceus* TL-3

Variables	Coded symbol	Coded values				
		− α	− 1	0	+ 1	+ α
Molasses	A	27.84	29.22	30.60	31.98	33.36
Meat extract	B	16	20	24	28	32
(NH_4_)_2_SO_4_	C	6.7	7.55	8.4	9.25	10.10
MnSO_4_	D	0.067	0.082	0.096	0.110	0.124

**Table 10:** CCD matrix with coded value and their corresponding experimental and predicted net threonine produced by *P. pentosaceus* TL-3

Std run	A	B	C	D	Net threonine produced (mg/L)	
					Experimental	Predicted^a^
1	− 1	− 1	− 1	− 1	71.90 ± 0.19^L^	71.79
2	1	− 1	− 1	− 1	75.28 ± 0.19^K^	75.95
3	− 1	1	− 1	− 1	76.98 ± 0.24^K^	77.71
4	1	1	− 1	− 1	84.67 ± 0.50^H^	83.27
5	− 1	− 1	1	− 1	79.72 ± 0.87^IJ^	80.07
6	1	− 1	1	− 1	85.62 ± 0.20^H^	83.83
7	− 1	1	1	− 1	89.11 ± 0.41^G^	92.59
8	1	1	1	− 1	98.25 ± 0.41^D^	97.75
9	− 1	− 1	− 1	1	75.87 ± 0.30^K^	75.07
10	1	− 1	− 1	1	81.66 ± 0.09^I^	79.19
11	− 1	1	− 1	1	82.09 ± 0.06^I^	84.91
12	1	1	− 1	1	92.07 ± 0.42^F^	90.43
13	− 1	− 1	1	1	77.48 ± 0.11^JK^	79.87
14	1	− 1	1	1	85.61 ± 0.09^H^	83.59
15	− 1	1	1	1	98.27 ± 0.29^D^	96.31
16	1	1	1	1	100.29 ± 0.17^D^	101.43
17	− 2	0	0	0	84.58 ± 0.46^H^	80.99
18	2	0	0	0	86.42 ± 0.26^H^	90.27
19	0	− 2	0	0	67.87 ± 0.13^M^	69.59
20	0	2	0	0	94.80 ± 0.24^E^	93.35
21	0	0	− 2	0	64.24 ± 0.15^N^	65.19
22	0	0	2	0	85.14 ± 0.16^H^	84.47
23	0	0	0	− 2	93.91 ± 0.13^EF^	93.03
24	0	0	0	2	98.88 ± 0.22^D^	99.99
25	0	0	0	0	118.84 ± 1.58^C^	121.27
26	0	0	0	0	118.93 ± 0.79^C^	121.27
27	0	0	0	0	124.95 ± 2.26^A^	121.27
28	0	0	0	0	122.01 ± 2.60^AB^	121.27
29	0	0	0	0	120.24 ± 1.55^BC^	121.27
30	0	0	0	0	122.65 ± 1.25^AB^	121.27
MRS	–	–	–	–	60.47 ± 0.72^O^	–

Values are mean ± standard error of mean (SEM), n = 3. Mean ± SEM within the same column that share different superscript are significantly different (p < 0.05)

^a^Predicted net threonine produced was calculated based on Eq. 3

The ANOVA of the regression models for threonine production by *P. pentosaceus* TL-3 is shown in Table 11. Amongst the 4 tested models, the data were significantly (p < 0.05) best fitted to a quadratic polynomial model, whereas the linear, crossproduct and cubic models were not significant (p > 0.05). Moreover, high adjusted R^2^ value (0.9744) and predicted R^2^ value (0.9379) were observed in the quadratic model with insignificant lack of fit (p > 0.05). This indicated that the high predictive strength and goodness of fit of the model. The high predictive strength of the model was further supported by the closeness between the experimental and predicted net threonine produced as shown in Table 10. Furthermore, the quadratic model was not aliased. Hence, the quadratic model was chosen as the best model to represent the threonine production by *P. pentosaceus* TL-3.

**Table 11:** ANOVA of regression model for threonine production by *P. pentosaceus* TL-3

Source	Sequential p-value	Lack of fit p-value	Adjusted R-squared	Predicted R-squared	
Linear	0.2610	0.0001	0.0530	0.0166	
Crossproduct	0.9998	< 0.0001	− 0.2333	− 0.3772	
Quadratic	< 0.0001	0.3344	0.9744	0.9379	Suggested
Cubic	0.1724	0.6420	0.9839	0.9043	Aliased

The effects of molasses, meat extract, (NH_4_)_2_SO_4_ and MnSO_4_ on the net threonine production by *P. pentosaceus* TL-3 (Y) can be expressed by Eq. (3) with coded symbols (A–D) as shown in Table 9.

3 $$\begin{aligned} {\text{Y }} &= 121.27 + 2.37{\text{A }} + 5.94{\text{B }} + 4.82{\text{C }} + 1.74{\text{D }} + 0.35{\text{AB }} - 0.1{\text{AC }} \hfill \\ & \quad - 0.012{\text{AD}} + 1.65{\text{BC }} + 0.98{\text{BD }} - 0.87{\text{CD }} - 8.91{\text{A}}^{2} - 9.95{\text{B}}^{2} \hfill \\ & \quad - 11.61{\text{C}}^{2} - 6.19{\text{D}}^{2} \hfill \\ \end{aligned}$$

The adequacy of quadratic model and significance of the variables on threonine production by *P. pentosaceus* TL-3 were evaluated by ANOVA (Table 12). The p-value of the model was less than 0.01, inferring that the model was highly significant and the F-value of the model that could be due to noise was less than 1%. In addition, the coefficient of determination, R^2^ of the model was 0.9868, implying that the model is capable of explaining 98.7% of the variation in response. Moreover, the predicted R^2^ (0.9379) was in reasonable agreement with the adjusted R^2^ (0.9744) and both R^2^ values were very close to 1. Moreover, the p-value of lack of fit (0.33) implied that the lack of fit was not significant, reflecting the high predictive strength of the model and great correlation between the experimental and predicted values [28]. Additionally, the adequate precision value of the model (28.578) was greater than 4, indicating an adequate signal compared to noise and the model could be used to navigate the design space. Based on Table 12, the linear coefficients (A, B, C, D) and quadratic coefficients (A^2^, B^2^, C^2^, D^2^) were highly significant (p < 0.01), while one of the interaction coefficient (BC) was significant at p-value less than 0.05.

**Table 12:** ANOVA for quadratic model of net threonine produced by *P. pentosaceus* TL-3

Source	Sum of squares	df	Mean square	F value	p-value Prob > F	
Model	8625.09	14	616.08	79.98	< 0.01	Significant
A	129.29	1	129.29	16.79	< 0.01	Significant
B	845.61	1	845.61	109.78	< 0.01	Significant
C	557.25	1	557.25	72.35	< 0.01	Significant
D	72.66	1	72.66	9.43	< 0.01	Significant
AB	1.97	1	1.97	0.26	0.62	
AC	0.17	1	0.17	0.022	0.88	
AD	2.275E−3	1	2.275E−3	2.953E−4	0.99	
BC	43.61	1	43.61	5.66	0.03	Significant
BD	15.22	1	15.22	1.98	0.18	
CD	12.08	1	12.08	1.57	0.23	
A^2^	2176.96	1	2176.96	282.63	< 0.01	Significant
B^2^	2715.77	1	2715.77	352.58	< 0.01	Significant
C^2^	3698.22	1	3698.22	480.13	< 0.01	Significant
D^2^	1049.34	1	1049.34	136.23	< 0.01	Significant
Residual	115.54	15	7.70			
Lack of Fit	87.06	10	8.71	1.53	0.33	Not significant
Pure Error	28.48	5	5.70			
Cor Total	8740.62	29				

R^2^: 0.9868; Adj R^2^: 0.9744; Pred R^2^: 0.9379; Adeq precision: 28.578

**Fig. 3:**
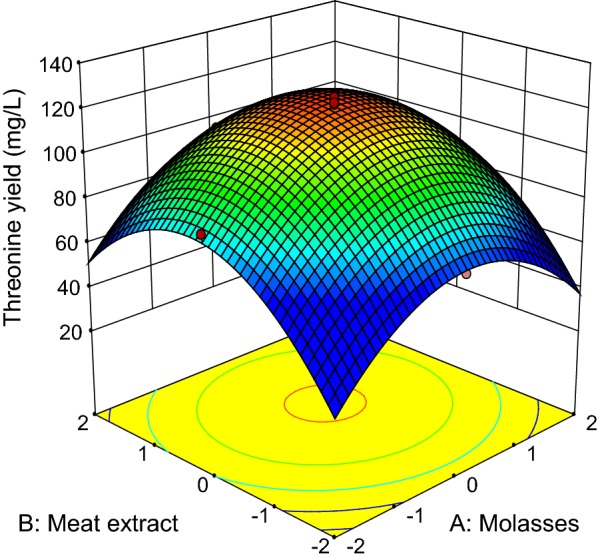
Response surface of net threonine produced by *P. pentosaceus* TL-3 as functions of molasses and meat extract

**Fig. 4:**
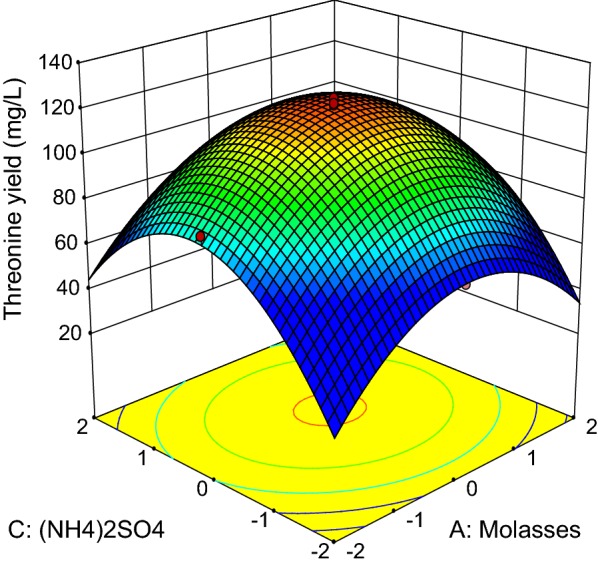
Response surface of net threonine produced by *P. pentosaceus* TL-3 as functions of molasses and (NH_4_)_2_SO_4_

**Fig. 5:**
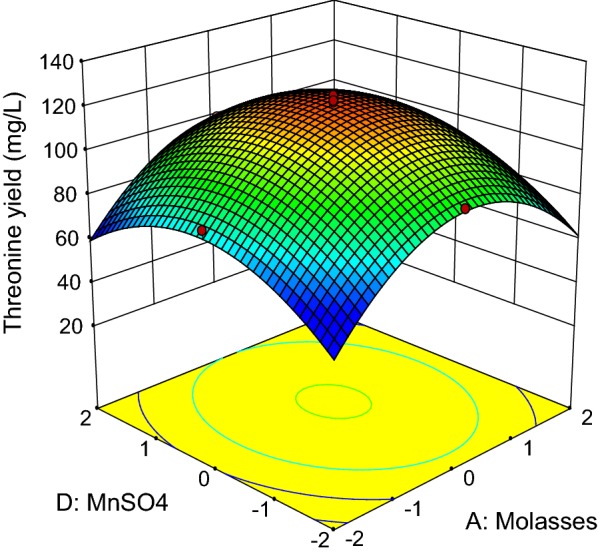
Response surface of net threonine produced by *P. pentosaceus* TL-3 as functions of molasses and MnSO_4_

**Fig. 6:**
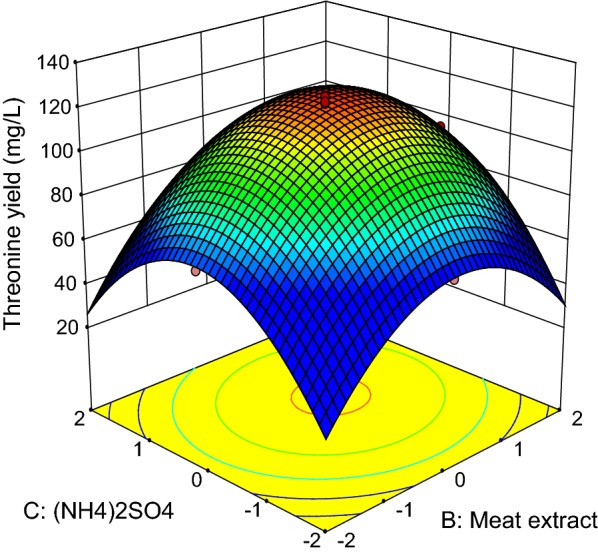
Response surface of net threonine produced by *P. pentosaceus* TL-3 as functions of meat extract and (NH_4_)_2_SO_4_

**Fig. 7:**
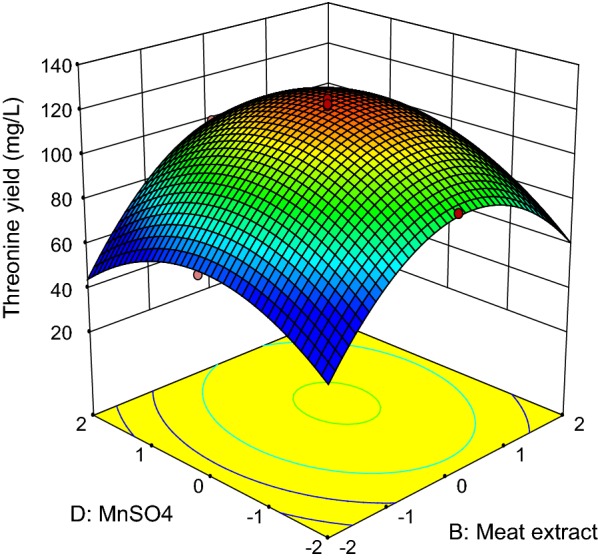
Response surface of net threonine produced by *P. pentosaceus* TL-3 as functions of meat extract and MnSO_4_

**Fig. 8:**
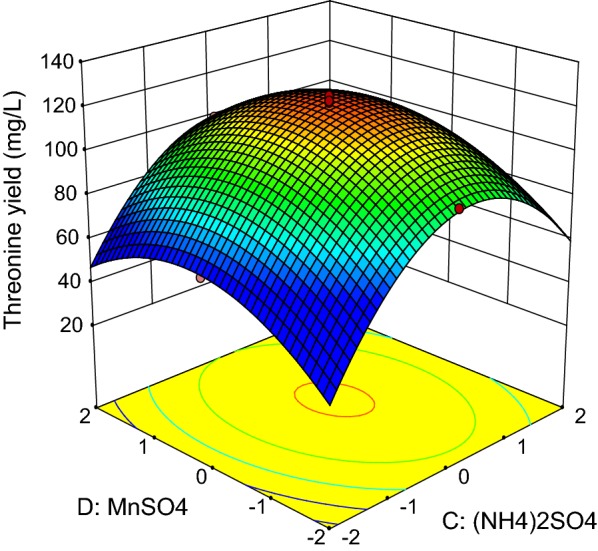
Response surface of net threonine produced by *P. pentosaceus* TL-3 as functions of (NH_4_)_2_SO_4_ and MnSO_4_

The three-dimensional response surface curves (Figs. 3, 4, 5, 6, 7 and 8) were subsequently plotted to illustrate the interactions between the medium components graphically. Generally, the highest net threonine produced was detected when the level of each medium component was near the center point. Figure 3 depicts the three-dimensional plot of threonine production by *P. pentosaceus* TL-3 as the functions of molasses and meat extract, whereby the (NH_4_)_2_SO_4_ and MnSO_4_ were fixed at the center point (8.4 g/L, 0.096 g/L). The maximum response was detected when the level of both molasses and meat extract was near 0. Increasing or decreasing the concentration of any of these medium components reduced the net threonine produced. Based on the p-value of the ANOVA analysis (Table 12), it implied that there were no significant interactions (p > 0.05) between the 2 variables.

Figure 4 illustrates the combined effects of molasses and (NH_4_)_2_SO_4_ on threonine production by *P. pentosaceus* TL-3, whereby the concentrations of meat extract and MnSO_4_ were kept at the center point (24 g/L, 0.096 g/L). Based on the ANOVA result (Table 12), both molasses and (NH_4_)_2_SO_4_ affected threonine production significantly (p < 0.05). However, the interaction between the two variables did not contribute significantly to the production of threonine by *P. pentosaceus* TL-3 (p > 0.05) and the highest net threonine produced was detected when the level of both variables was near the center point. Meanwhile, Fig. 5 displays the three dimensional surface plot as functions of molasses and MnSO_4_, whereby the concentrations of meat extract and (NH_4_)_2_SO_4_ were fixed at the center point (24 g/L, 8.4 g/L). Similarly, the interaction between the 2 variables was not significant (p > 0.05) and the highest response was observed when both variables were in the range of − 1 to + 1.

On the other hand, the interaction between meat extract and (NH_4_)_2_SO_4_ is shown in Fig. 6, whereby the concentrations of molasses and MnSO_4_ were set at the center point (30.60 g/L, 0.096 g/L).The highest net threonine produced was detected when the level of meat extract was between 0 and + 1, while the concentration of (NH_4_)_2_SO_4_ was kept near to the center point. Based on the ANOVA results (Table 12), the interaction effect between meat extract and (NH_4_)_2_SO_4_ was significant at p-value less than 0.05. Figure 7 depicts the three dimensional surface plot of net threonine produced as a function of meat extract and MnSO_4_, where the level of molasses and (NH_4_)_2_SO_4_ were kept at the center point (30.60 g/L, 8.4 g/L). The highest net threonine produced was observed when the level of meat extract was between 0 and + 1, while the level of MnSO_4_ was varied between − 1 and + 1. However, the ANOVA results (Table 12) demonstrated that the interaction between meat extract and MnSO_4_ did not contribute significantly (p > 0.05) to the production of threonine by *P. pentosaceus* TL-3.

The combined effects of (NH_4_)_2_SO_4_ and MnSO_4_ on threonine production by the producer strain was illustrated in Fig. 8, whereby the levels of molasses and meat extract were fixed at the center point (30.60 g/L, 24 g/L). The highest threonine production was detected when the levels of (NH_4_)_2_SO_4_ and MnSO_4_ were near the center point. Nevertheless, ANOVA analysis (Table 12) showed that the interaction effect between (NH_4_)_2_SO_4_ and MnSO_4_ was not significant (p > 0.05). The optimized level of each medium components was then computed by using Design Expert statistical software version 9.0.6.2 (State-Ease Inc, Minneapolis) with the setting of all medium components at the range of − 1 to + 1 and maximum response. By taking into consideration of these criteria, the optimum concentration of molasses, meat extract, (NH_4_)_2_SO_4_ and MnSO_4_ were found to be 30.79 g/L, 25.30 g/L, 8.59 g/L, and 0.098 g/L respectively with a predicted net threonine production of 123.07 mg/L. Validation of the statistical model was performed by cultivating *P. pentosaceus* TL-3 in the suggested optimized medium and an amount of 125.98 mg/L net threonine was produced. The production of threonine by *P. pentosaceus* TL-3 in the optimized medium was enhanced approximately by 2 folds as compared to MRS medium.

## Conclusions

Molasses, meat extract, (NH_4_)_2_SO_4_ and MnSO_4_ were identified to be the most essential medium components for threonine production by *P. pentosaceus* TL-3 via PBD and selected for further optimization. The optimum operating region of each medium component was determined by using steepest ascent method. The highest net threonine production of 116.83 mg/L was detected in run 5. Further increment of the concentrations of medium components reduced the net threonine produced. Thus, the operating parameters of run 5 was selected as center point for further optimization in CCD. The optimum concentration of molasses, meat extract, (NH_4_)_2_SO_4_ and MnSO_4_ were found to be 30.79 g/L, 25.30 g/L, 8.59 g/L, and 0.098 g/L respectively, based on the model obtained in CCD with a predicted net threonine production of 123.07 mg/L. The statistical model was subsequently validated by cultivating the producer strain in the optimized medium and up to 125.98 mg/L of net threonine produced was detected, which was not significantly different (p > 0.05) as compared to the predicted amount. The net threonine produced by *P. pentosaceus* TL-3 in the optimized medium was enhanced approximately by 2 folds in comparison to the control MRS medium through RSM optimization approach.

## Materials and methods

### Microorganism and inoculum preparation

The threonine producing LAB employed in current study, *P*. *pentosaceus* TL-3, was previously isolated from Malaysian fermented food, *Tempeh* [82]. The bacterial strain was obtained from the Laboratory of Industrial Biotechnology, Department of Bioprocess Technology, Faculty of Biotechnology and Biomolecular Sciences, Universiti Putra Malaysia. The culture was grown in de Man, Rogosa and Sharpe (MRS) medium (Merck, Germany) and preserved in MRS medium supplemented with 20% (v/v) glycerol (Merck, Germany) at − 20 °C as described by Kareem et al. [83]. The active culture was washed once with sterile 0.85% (w/v) NaCl (Merck, Germany) solution and adjusted to 10^9^ CFU/mL prior to use as inoculum for the experiment [84].

### Experimental design

The effects of medium components on threonine production by *P. pentosaceus* TL-3 were first evaluated by using PBD. Subsequently, the effects of significant variables identified in the PBD were validated and the medium formulation that showed the highest threonine production was selected for further optimization. Next, the steepest ascent method was employed to search for the vicinity of optimum operating regions for threonine production by *P. pentosaceus* TL-3. Thereafter, the optimum level of each medium component for threonine production by *P. pentosaceus* TL-3 was determined by using CCD, followed by the validation of threonine production by *P. pentosaceus* TL-3 using the optimized medium.

### Plackett Burman design

PBD was used to evaluate the effects of medium components on threonine production by *P. pentosaceus* TL-3 [85]. The design of experiment and statistical analysis of data were performed by using Design Expert statistical software version 9.0.6.2 (State-Ease Inc, Minneapolis). A total of 22 medium components including 5 carbon sources (glucose, sucrose, fructose, lactose and molasses); 3 organic nitrogen sources (yeast extract, peptone and meat extract); 4 inorganic nitrogen sources (urea, NH_4_NO_3_, (NH_4_)_2_SO_4_ and (NH_4_)_2_HC_6_H_5_O_7_); 8 mineral sources (K_2_HPO_4_, KH_2_PO_4_, NaOAc, MgSO_4_, MnSO_4_, FeSO_4_, ZnSO_4_ and CuSO_4_); 1 non-ionic surfactant (Tween 80) and 1 vitamin (biotin), which may have effect on amino acid production were selected by referring to the MRS medium compositions and published reports. Each of the selected medium component was assigned at 2 levels, which were high level (+ 1) and low level (− 1) as shown in Table 1. A 24 experimental runs PBD was suggested by the software as presented in Table 2. The response was expressed by the first-order model as shown in Eq. (4):

4 $$\text{Y} =\upbeta_{0} + \mathop \sum \limits_{i = 1}^{22}\upbeta _{\text{i}} \text{X}_{\text{i}}$$

where *Y* represents the response variable, *ß*_0_ is the interception coefficient and *ß*_i_ is the coefficients of the linear effects of the 22 independent variables (*X*_1_ − *X*_22_).

A validation test was subsequently conducted to verify the effect of the significant medium components identified in the PBD on threonine production by *P. pentosaceus* TL-3. A total of 7 media (Table 5) were formulated based on the ANOVA results of threonine production obtained from the PBD study (Table 3). Formulation 1 comprised all the significant medium components; Formulation 2 contained only the significant medium components with positive effect, whereas Formulation 3 was similar with Formulation 2 with addition of molasses since it was the only carbon source exerted a positive effect. In comparison, Formulation 4 was made up of all the medium components with positive effect regardless of their level of significance; Formulation 5, 6 and 7 constituted of 4 main medium components representing the carbon source, organic nitrogen source, inorganic nitrogen source and mineral source. The basal medium of Formulation 5, 6 and 7 contained molasses, (NH_4_)_2_SO_4_ and MnSO_4_ as carbon source, inorganic nitrogen source and mineral source respectively. However, they varied in organic nitrogen source, whereby Formulation 5, 6 and 7 contained peptone, meat extract and yeast extract respectively.

### Steepest ascent method

The steepest ascent method was used to determine optimum concentration proximity of the significant medium components for threonine production by *P. pentosaceus* TL-3. Based on the results obtained in validation test of PBD, four medium components [molasses, meat extract, (NH_4_)_2_SO_4_ and MnSO_4_] that gave the highest net threonine production were selected for further optimization. The first-order model generated by the PBD (Eq. 1) was employed to determine the direction and step length of each medium component in the steepest ascent method. The largest coefficient was used as the standard for the calculation of step length [86]. All tested medium components carried a positive sign and moved along the path of steepest ascent. The concentration of molasses, (NH_4_)_2_SO_4_ and MnSO_4_, was increased by 5.5%, 17% and 34% respectively, when the concentration of meat extract was increased by 50%. The steepest ascent design for threonine production by *P. pentosaceus* TL-3 is presented in Table 7. The medium that resulted the highest net threonine produced was selected for further optimization by CCD.

### Central composite design

The optimum concentration of the selected medium components (molasses, meat extract, (NH_4_)_2_SO_4_ and MnSO_4_) for threonine production by *P. pentosaceus* TL-3 was determined by using CCD. The design of experiment and statistical analysis of the data were performed by using Design Expert statistical software version 9.0.6.2 (State-Ease Inc, Minneapolis, MN). The concentration of each medium component was assigned to 5 levels: high level (+ 1), low level (− 1), central point (0) and 2 axial points (± α) as shown in Table 9. An axial distance of 2 was chosen to make the design rotatably. The CCD suggested a total of 30 experimental runs (16 factorial points, 8 axial points and 6 central points) as shown in Table 10. The production of threonine by *P. pentosaceus* TL-3 was expressed by the second-order model as shown in Eq. (5):

5 $$Y = \beta_{0} + \sum \beta_{j} X_{j} + \sum \beta_{{j^{2} }} X_{{j^{2} }} + \sum \beta_{jk} X_{j} X_{k}$$

where *Y* denotes the response variable and *ß*_0_ is the interception coefficient, while *ß*_j_, *ß*_j_^2^, and *ß*_jk_ were linear, quadratic and interactive coefficient respectively.

### Production of threonine

The extracellular production of threonine was conducted as described by Lim et al. [26]. A volume of 10% (v/v) inoculum prepared as described in “Microorganism and inoculum preparation” section was inoculated into the designed media and incubated at 30 °C for 10 h. The cultured broth was centrifuged at 10,000×*g* for 10 min at 4 °C to separate the biomass from the supernatant. The biomass was used for the determination of cell population, whereas the supernatant was used for the determination of threonine production.

## Analytical methods

### Cell population determination

The cell pellet was washed once with 0.85% (w/v) NaCl solution, followed by a 10-fold serial dilution from 10^0^ to 10^−9^. Next, 100 µL of each dilution was spread on MRS agar plate and incubated for 48 h at 30 °C. The cell population was calculated by using the following equation:

$${\text{Cell population }}\left( {{\text{log CFU}}/{\text{mL}}} \right) = \log \frac{\text{Colony forming unit}}{{{\text{Dilution factor }} \times {\text{Volume of culture }}\left( {\text{mL}} \right)}}$$

### Threonine concentration determination

The extracellular threonine content was determined by using Agilent 1260 high performance liquid chromatograph (HPLC) (Agilent Technologies, USA) as described by Henderson et al. [17, 87]. Derivatization of amino acids were performed by using o-phthalaldehyde (OPA) and 9-fluorenylmethyl chloroformate (FMOC). The derivatized amino acids were separated on a Zorbax Eclipse Plus C18 reverse phase column (4.6 mm × 150 mm, 3.5 µm) (Agilent Technologies, USA), followed by elution with 40 mM sodium dihydrogen phosphate monohydrate (Merck, Germany) pH 7.8 buffer and a methanol–acetonitrile-deionized water mixture (9:9:2) at a constant flow rate of 2 mL/min. The OPA, FMOC and NaH_2_PO_4_·H_2_O were analytical grade, while the methanol and acetonitrile were HPLC grade that purchased from Merck. The eluted derivatized amino acids were detected by a fluorescence detector at the excitation/emission wavelengths of 340/450 nm. The threonine concentration was quantified by referring to the calibration curve constructed by using amino acid standard (Sigma Aldrich, USA). The production of threonine was calculated by deducting the final concentration of threonine for each experimental run with their respective initial threonine concentration. All analyses were performed in triplicates.

### Statistical analysis

The results were analyzed by one-way analysis of variance (ANOVA) using Statistical Analysis System (SAS 9.1, USA). Duncan’s Multiple Range Test System was used to compare the significant difference between the mean at p < 0.05.

## Data Availability

The datasets used and/or analysed during this study are available from the corresponding author on reasonable request.

## Abbreviations

LAB: lactic acid bacteria
GRAS: Generally Recognized as Safe
EMS: eosinophils myalgia syndrome
PBD: Plackett–Burman design
CCD: central composite design
NADP^+^: nicotinamide adenine dinucleotide phosphate
GABA: gamma-aminobutyric acid
ANOVA: analysis of variance
MRS: de Man, Rogosa and Sharpe
HPLC: high performance liquid chromatograph
OPA: *o*-phthalaldehyde
FMOC: 9-fluorenylmethyl chloroformate
